# SPARC Levels Modulate the Capacity of Mitomycin to Inhibit the Proliferation of Human Tenon's Capsule Fibroblasts

**DOI:** 10.1155/2020/5703286

**Published:** 2020-02-10

**Authors:** Yuanyuan Guo, Shouxiang Ni, Weiyan Zhou, Jiangping Hou, Jiaquan Shen

**Affiliations:** Department of Ophthalmology, Shandong Provincial Hospital Affiliated to Shandong University, Jinan, Shandong, China

## Abstract

**Purpose:**

To evaluate the role of SPARC in the antiproliferation effect of MMC on human Tenon's fibroblasts (HTF).

**Method:**

Sixteen PACG patients aged 59 ± 10 years (31–72 years), including 6 males and 10 females, were recruited. Tenon tissue was harvested during filtering surgery. Cell density was evaluated after MMC application with different concentrations and application times, by which the optimized MMC application modality was determined. MMC, si-SPARC, or SPARC protein was used when needed to evaluate the cell densities under different conditions, by which the role of SPARC in MMC-mediated antifibrotic process was identified.

**Results:**

Considering that the cell densities, as well as SPARC expression on mRNA and protein levels, are relatively stable when the MMC concentration is higher than 0.02% and exposure time longer than 90 s, we chose the MMC application pattern with 0.02% and 90 s as an optimized pattern for the downstream work. Compared to control, the si-SPARC and MMC downregulated the SPARC protein by 91% (*P* < 0.01) and 65% (*P* < 0.01) and 65% (*P* < 0.01) and 65% (*P* < 0.01) and 65% (*P* < 0.01) and 65% (*P* < 0.01) and 65% (*P* < 0.01) and 65% (*P* < 0.01) and 65% (

**Conclusion:**

This study demonstrates that in HTF, (1) MMC downregulates the expression of SPARC in protein and mRNA levels; (2) SPARC depletion has synergistic effect on the antifibrotic effect of MMC; and (3) reactive oxygen species are the possible mediator in the antifibrotic effect of MMC and si-SPARC.

## 1. Introduction

Trabeculectomy was first introduced by Cairns in 1968 [1] and has been a widely used therapy strategy for glaucoma. A successful bleb and efficient filtration are the foundation of sound outcome. The excessive proliferation of fibroblast and the formation of scar of Tenon's capsule are the major reasons for the bleb failure.

Mitomycin (MMC), as a commonly used antimetabolism drug during trabeculectomy, has a pronounced effect on inhibiting fibroblast proliferation and scar formation [2]. MMC application modality is highly related to the success rate of bleb. Nonetheless, the MMC application modality should ideally be personally modulated according to the preoperative IOP, anterior chamber depth, and the thickness of Tenon's capsule. Although MMC is considered beneficial to the clinical outcome of trabeculectomy, the side effects of MMC should be considered during application. Previous studies have reported the side effects of mitomycin that include conjunctival atrophy, inflammation, and cytological atypia [3, 4], persistent decrease in keratocyte density [5], and corneal endothelium damage [6]. It is interesting to investigate how to achieve the reasonable antifibrotic effect with a lower MMC concentration, which may provide a possible means to decrease the risks of MMC-related side effects.

Secreted protein acidic and rich in cysteine (SPARC) plays an important role in the occurrence of primary angle-closure glaucoma (PACG) [7]. Previous studies have verified SPARC is a key mediator and indicator for the antiproliferation effect of MMC [8] and can improve the success rate of filtering surgery [9]. The present study evaluates the SPARC expression variation during MMC application on human Tenon's fibroblasts (HTF) and explores the way of enhancing the antifibrotic effect of MMC on HTF with si-RNA technique, by which a therapy strategy with lower concentration MMC may be available under the combination usage of si-SPARC technique.

## 2. Methods

### 2.1. Study Subjects

The present study enrolled 16 (6 male and 10 female) patients with PACG, aged 59 ± 10 years (range, 31–72 years), that were admitted to Shandong Provincial Hospital between December 2017-2018. Patients with a history of infectious or noninfectious eye inflammation, systemic immune disorders, and/or scar diathesis were excluded. All subjects provided informed consent for their participation in the study, which was approved by the Ethical Committee of the Shandong Provincial Hospital and conducted in accordance with the tenets of the Declaration of Helsinki and with the National Drug Law of China.

### 2.2. HTF Isolation and Treatment

Sections (2 × 3 mm^2^) of Tenon's capsule tissue were harvested from each patient during glaucoma filtration surgery and cut into six 1 × 1 mm^2^ tissue blocks. They were washed thrice with PBS and seeded into 6-well plates. Cells were maintained (37°C, 5% CO_2_) in fibroblast culture medium (Cat. No. 2301, Zhong Qiao Xin Zhou Co. Ltd. China). After 3 days of incubation, the cells were harvested and subcultured in 24-well plates. After incubation for 1 day, cell attachment was observed, and the cells were treated with 0.01%, 0.02%, or 0.04% MMC solution in phosphate-buffered saline (PBS) for 30, 60, 90, or 120 s and/or transfected with a previously designed siRNA targeted to *SPARC* (100 nM; *si-SPARC*, 5′-AACAAGACCUUCGACUCUUCC-3′) [8], or a scrambled control siRNA (100 nM; *si-Scram*, 5′-GCUCACAGCUCAAUCCUAAUC-3′; synthesized and purified by Bioneer, General Biosystems, China), using Lipofectamine 2000 (ThermoFisher Scientific Inc. USA) according to the instructions of the manufacturer. Cells after treatment were harvested on day 3 for future use.

### 2.3. HTF Proliferation Analysis

Cell counts were performed (in triplicate) using a Cell Counting Chamber Slide (Thermo Fisher Scientific Inc. USA), and all values were expressed relative (i.e., as a ratio) to the final density of control (untreated) cells (2 × 10^4^ cells/mL).

### 2.4. Collagen Gel Contraction Assays

The collagen contraction assays were performed as previously described [9]. Cells were seeded in a collagen type-I matrix (First Link Ltd., Birmingham, UK) at a concentration of 50,000 cells/mL. The gels were detached from the edge of the well with a microspatula. Gels were incubated for two days, and then digital photographs were obtained daily over 7 days. Gel areas were measured using ImageJ software (version for 64 bit Windows, USA.), and the contraction ratio was expressed as a percentage of gel area normalized to day 0.

### 2.5. Western Blot Analysis

Total cellular extracts were prepared by lysing cells in lysate solution (HConF Lysate, Zhong Qiao Xin Zhou Co. Ltd., China) and subjected to a western blot analysis, including SDS-PAGE, immunoblotting, and densitometric quantitation, as previously described [8]. Antibodies against SPARC and *β*-actin were purchased from Thermo Fisher Scientific Inc. (USA). Data were expressed relative to control SPARC expression levels. SPARC expression levels were normalized to that of *β-actin*, and generated data were expressed relative to controls.

### 2.6. Quantitative Real-Time PCR (qRT-PCR) Analysis

Previously described methods were used to extract the total RNA from HTFs, synthesize cDNA, and conduct qRT-PCRs [10]. All reactions were performed in triplicate, using primers targeting *SPARC* (forward, 5′TTGCCTGAGGCTGTAACTGA3′; reverse, 5′GGGAGGGTGAAGAAAAGGAG3′), and *β-actin* (forward, 5′GGCTACAGCTTCACCACCAC3′; reverse, 5′AGGAAGGAAGGCTGGAAGAG3′). The *SPARC* cycle threshold (CT) value was normalized to that of *β-actin*, and generated data were expressed relative to controls.

### 2.7. Reactive Oxygen Species (ROS) Analysis

Cells were incubated for 24 h in the absence (control) or in the presence of MMC or si-SPARC.

Analysis was performed using DCFDA Cellular ROS Detection Assay Kit (Abcam, UK) according to the product manual and previous report [11]. Cells were seeded into a 96-well microplate with 25,000 cells per well and cultured overnight for cell adhering. The medium was washed with 1 × buffer, and the cells were stained by adding 100 *µ*L/well of the diluted DCFDA solution and incubated for 45 minutes at 37°C in the dark. DCFDA solution was removed and 100 *µ*L/well of 1 ×  buffer was added, and then fluorescence measurement was performed immediately. Measurement was performed on a fluorescence plate reader at *E*_x_/*E*_m_ = 485/535 nm using a Packard EL340 microplate reader (Bio-Tek Instruments, Winooski, VT).

### 2.8. Statistical Analysis

Data were statistically assessed using a Friedman or linear regression analysis, using MedCalc software (Windows version 15.2.2). *P* < 0.05 was considered to be statistically significant.

## 3. Results

A total of 16 patients aged 59 ± 10 years (31–72 years), including 6 males and 10 females, were involved in the present study.

**Figure 1:**
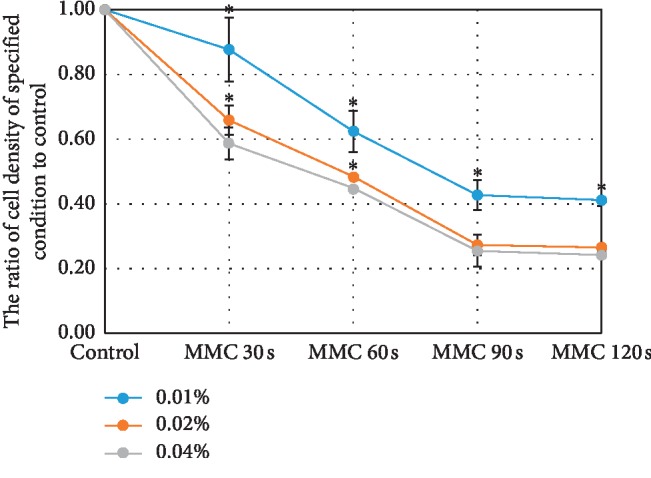
The cell densities in the 0.01% MMC group are higher than those in the 0.02% and 0.04% MMC groups under the condition of application time of 90 s (*P* < 0.05) or 120 s (*P* < 0.05). The cell densities in 4 conditions (0.02% + 90 s; 0.02% + 120 s; 0.04% + 90 s; 0.04% + 120 s) have no difference (*P* > 0.05). ^*∗*^Cell density in the specified group has a significant difference (*P* < 0.05) with that in the 0.04% MMC group, respectively.

Experiments (3 wells) were performed in triplicate for each experimental condition for each patient, in the procedure defining the optimizing MMC application modality. The cell densities in each well were counted 3 times. The cell density ratios in the MMC group with application time of 30 s, 60 s, 90 s, and 120 s were 0.88 ± 0.05, 0.63 ± 0.11, 0.43 ± 0.03, 0.41 ± 0.02 in the 0.01% MMC group, respectively, 0.66 ± 0.09, 0.49 ± 0.04, 0.27 ± 0.02, 0.27 ± 0.03 in the 0.02% MMC group, respectively, and 0.59 ± 0.06, 0.45 ± 0.04, 0.25 ± 0.04, 0.24 ± 0.02 in the 0.04% MMC group, respectively (Figure 1). The cell densities in the 0.01% MMC group were higher than those in the 0.02% and 0.04% MMC groups under the application time condition of 90 s or 120 s (*P* < 0.05). However, the cell densities in the 0.02% and 0.04% MMC group have no difference under the application time condition of 90 s or 120 s. In other words, the cell densities in 4 conditions (0.02% + 90 s; 0.02% + 120 s; 0.04% + 90 s; 0.04% + 120 s) have no difference (*P* < 0.05).

Based on the above data, we chose the MMC application pattern with 0.02% and 90 s with an optimized pattern for the downstream work.

**Figure 2:**
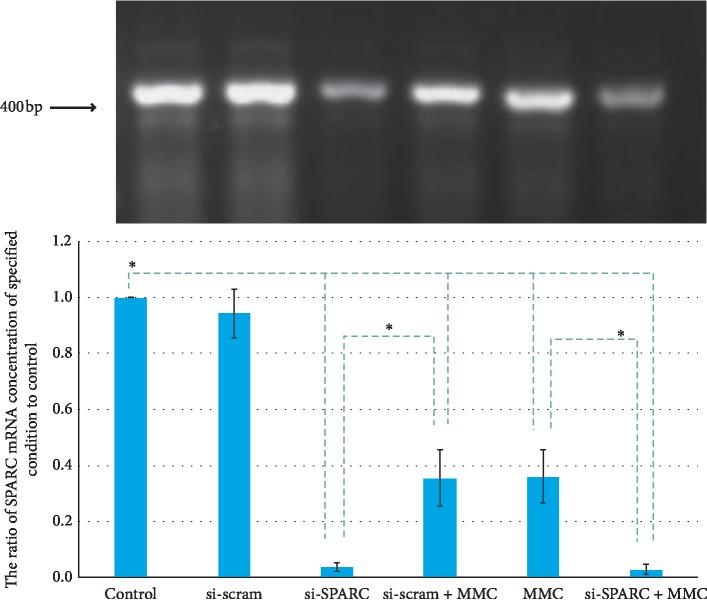
Under 0.02% MMC + 90 s condition, the si-SPARC and MMC downregulated the SPARC mRNA by 96% (*P* < 0.01) and 64% (*P* < 0.01), compared with control. ^*∗*^*P* < 0.05 compared with control, unless specified.

The SPARC mRNA expression ratios of the si-Scram group, si-SPARC group, si-Scram + MMC group, MMC group, and MMC + si-SPARC group were 0.94 ± 0.09, 0.04 ± 0.01, 0.35 ± 0.10, 0.36 ± 0.09, and 0.03 ± 0.02, respectively (Figure 2). Compared with control, the si-SPARC and MMC downregulated the SPARC protein by 96% (*P* < 0.01) and 64% (*P* < 0.01), respectively, which is consistent with their effect on SPARC protein expression.

**Figure 3:**
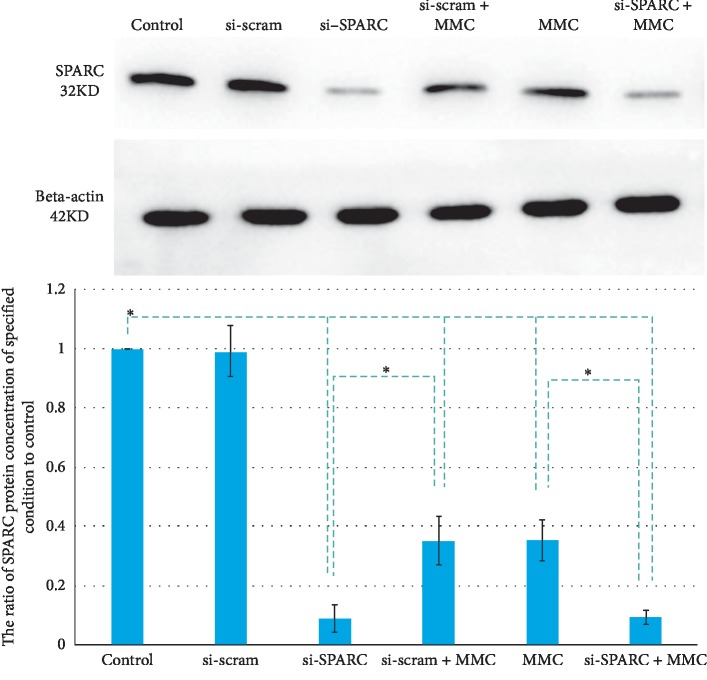
Under 0.02% MMC + 90 s condition, the si-SPARC and MMC downregulated the SPARC protein by 91% (*P* < 0.01) and 65% (*P* < 0.01), compared with control. ^*∗*^*P* < 0.05 compared with control, unless specified.

The SPARC protein expression ratios to reference protein of the si-Scram group, si-SPARC group, si-Scram + MMC group, MMC group, and MMC + si-SPARC group were 0.99 ± 0.08, 0.09 ± 0.05, 0.35 ± 0.08, 0.35 ± 0.07, and 0.09 ± 0.02, respectively (Figure 3). Compared with control, the si-SPARC and MMC downregulated the SPARC protein by 91% (*P* < 0.01) and 65% (*P* < 0.01), respectively.

**Figure 4:**
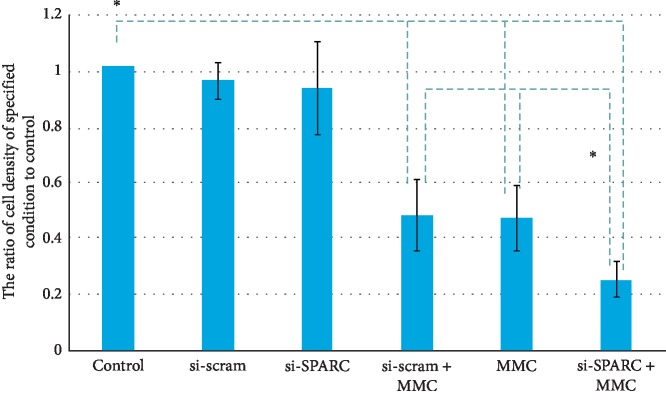
MMC decreases the cell densities by 53.50% compared to control. si-SPARC + MMC dramatically deceased the cell density no matter compared to the control group (*P* < 0.01) or MMC group (*P* < 0.01). ^*∗*^*P* < 0.05 compared with control, unless specified.

The cell density ratios of the si-Scram group, si-SPARC group, si-scram + MMC group, MMC group, and MMC + si-SPARC group were 0.95 ± 0.07, 0.92 ± 0.16, 0.48 ± 0.13, 0.47 ± 0.11, and 0.25 ± 0.06, respectively (Figure 4). si-SPARC does not impede the proliferation of HTF, and this result is consistent with the previous study [8]. MMC decreases the cell densities by 53.50% compared to control. Interestingly, si-SPARC + MMC dramatically deceased the cell density no matter compared to the control group (*P* < 0.01) or MMC group (*P* < 0.01). This result demonstrates a significant synergistic effect of si-SPARC on the antifibrotic effect of MMC on HTF proliferation.

**Figure 5:**
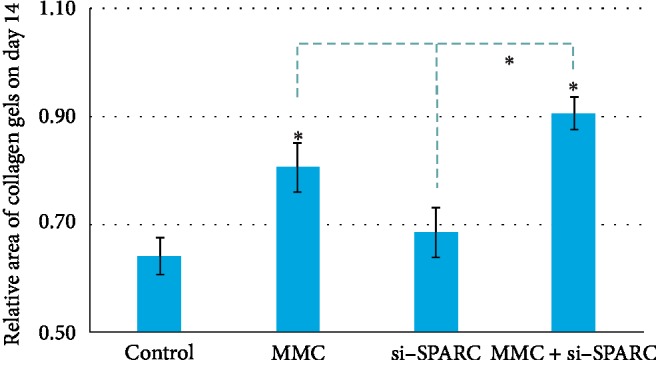
The relative gel area in the MMC + si-SPARC group is higher than that in the MMC group (*P* < 0.05). ^*∗*^*P* < 0.05 compared with control, unless specified.

**Figure 6:**
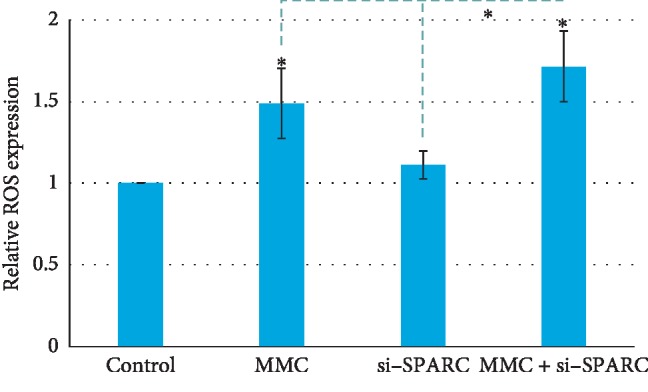
The ROS expression in the MMC + si-SPARC group is higher than that in the MMC group (*P* < 0.05). ^*∗*^*P* < 0.05 compared with control, unless specified.

The relative gel areas in the control group, MMC group, si-SPARC group, and MMC + si-SPARC group were 0.64 ± 0.03, 0.81 ± 0.05, 0.68 ± 0.05, and 0.91 ± 0.03, respectively. The relative gel area in the MMC + si-SPARC group is higher than that in the MMC group (*P* < 0.05) (Figure 5). Accordingly, the relative ROS expressions in the MMC group, si-SPARC group, and MMC + si-SPARC group were 1.41 ± 0.23, 1.19 ± 0.08, and 1.72 ± 0.15, respectively. The ROS expression in the MMC + si-SPARC group is higher than that in the MMC group (*P* < 0.05) (Figure 6).

## 4. Discussion

The effect of MMC on IOP reduction is related to the application time, area [12, 13], location [14], and concentration. The concentration and exposure duration are two main parameters modified in previous studies, in which the concentration ranged from 0.01% to 0.04% and exposure duration ranged from 1 minute to 5 minutes [15–20]. The previous study has shown that the bioactivity of MMC on fibroblasts is time-dependent; in detail, 60 s of exposure to 0.04% MMC is as effective as a 300 s exposure in vitro [21]. The present study showed the effect of MMC with 0.02% concentration and 90 s exposure time is comparable with that of MMC with higher concentration and longer exposure time, and the MMC with 0.02% concentration and 90 s exposure time was used as an optimized modality in the present study. The EC50 of MMC is 0.05% MMC under 30 s application time condition, which is consistent with the present data, in which the proportion to maximal effect under 0.04% MMC + 30 s condition is 48%.

SPARC also known as osteonectin and BM-40 is participated in the modulation of cell interactions [22]. SPARC interacts with a large number of crucial factors, such as matrix metalloproteinases (MMPs), by which it attains its bioactivity including ECM remodelling. However, the investigation of SPARC is still scarce considering its complicated interactions and bioactivities. Seet et al. showed the depletion of SPARC improved the success rate of filtering surgery [10], and the SPARC-null conjunctival tissue has a lower collagen I expression and a compromised maturation and assembly of the ECM after filtering surgery [10]; however, the underlying mechanism has not been elaborated.

The present result showed that MMC dramatically downregulated the expression of SPARC on mRNA and protein levels, and the SPARC deficiency condition cannot inhibit the proliferation of HTF, which is consistent with previous reports [10]. Present result also showed an observable synergistic effect of SPARC depletion on the antifibrotic effect of MMC, as evident by a 45.78% decrease of HTF cell density in the si-SPARC + MMC group than that in MMC group, and by a increase of relative gel area in the si-SPARC + MMC group than that in MMC group.

The present study showed that the application of MMC dramatically upregulates the expression of ROS, which is enhanced by the combined application of si-SPARC. The relation of SPARC and ROS production is rather complicated. Shibata has revealed SPARC is required for TGF-*β*-induced ROS production in A549 cell lines [23]; however, other evidence showed that the removal of SPARC is associated with augmented ROS accumulation [24] and induces a reduction of cell motility and collagen gel contraction in HTF [8].

The reduction in A549 cell viability was also alleviated in the presence of N-acetylcysteine (NAC) [25] NAC is a synthetic precursor of intracellular cysteine and thus is considered as an important antioxidant as ROS scavengers, especially considering cysteine is frequently observed in functionally important sites of proteins. Meduri demonstrated that all the eyes of patients treated with cysteine oral supplements had shorter times to corneal wound healing after photorefractive keratectomy, which verified the importance of cysteine during tissue recovery [26].

It is well known that SPARC contains 10 residues of cysteine and ROS are increasingly recognized as important signalling molecules through oxidation of protein cysteine residues of which oxidation changes the protein function, thus enabling signal transmission to downstream targets [27]. It is reasonable to speculate the cysteine residue of SPARC plays a certain role during the MMC + si-SPARC-induced antifibrosis process. Considering the oxidation of cysteine leads to a lack of protein function, the interaction of ROS and cysteine residues in SPARC during the MMC + si-SPARC-induced antifibrosis process should be further investigated in the future study.

To date, several studies have verified the feasibility of the in vivo application of the siRNA sequence on eye tissue [28, 29]. It is possible to apply silencing SPARC in vivo with a similar technique, and the in vivo application of silencing SPARC in patients receiving filtering surgery is an important researching field of our future study.

## Data Availability

The data used to support the study are available at http://wp.163.com/ (raw data can be obtained with user yuanyuanguo2 and password Ab123456).
